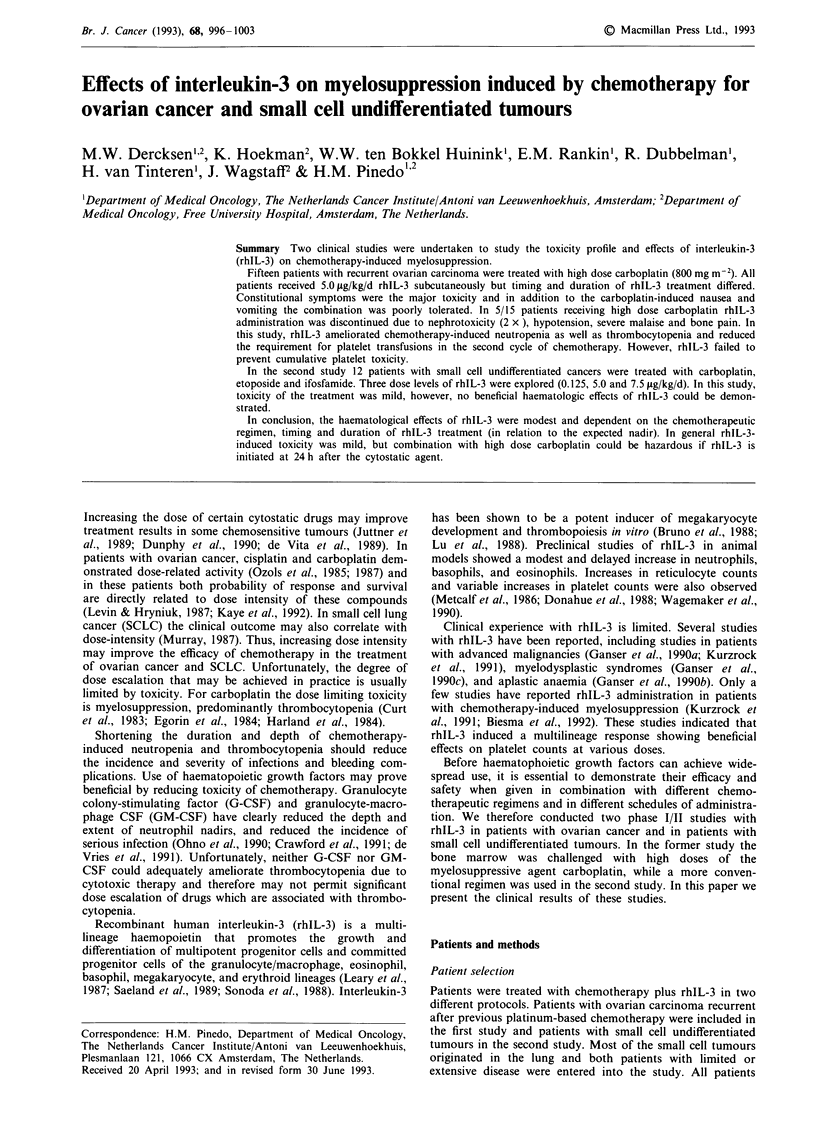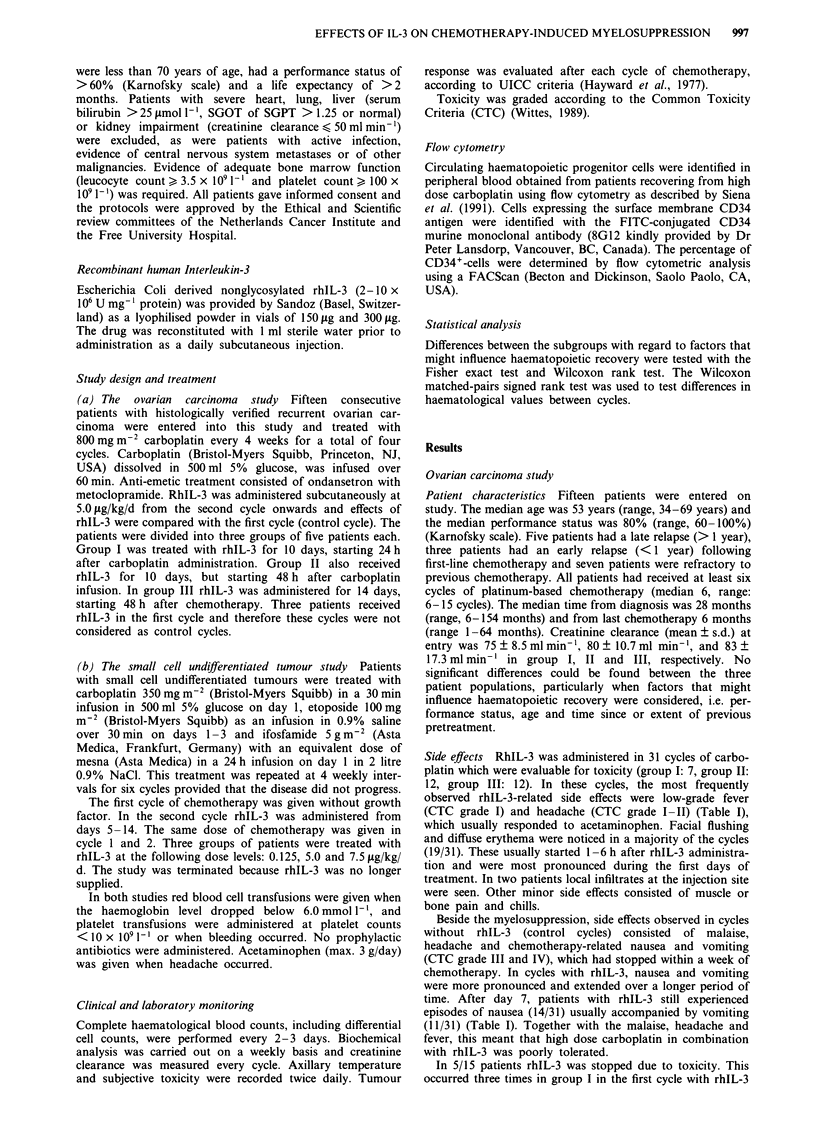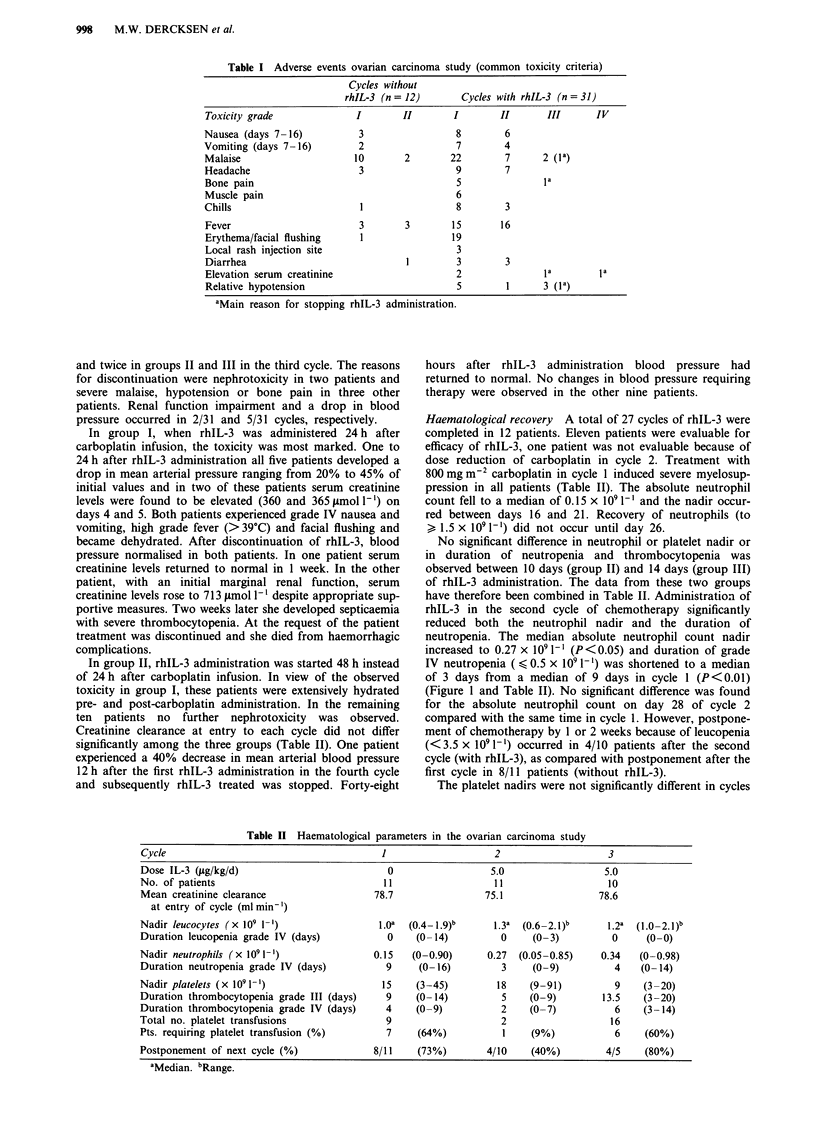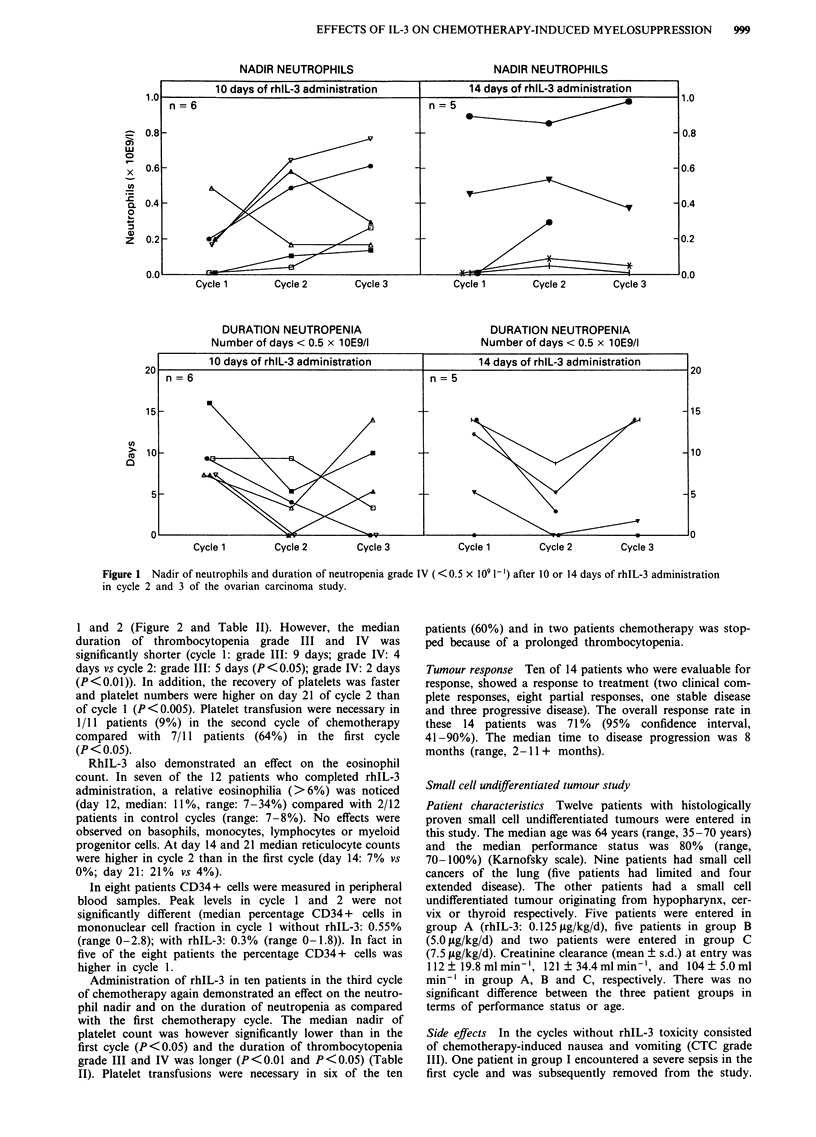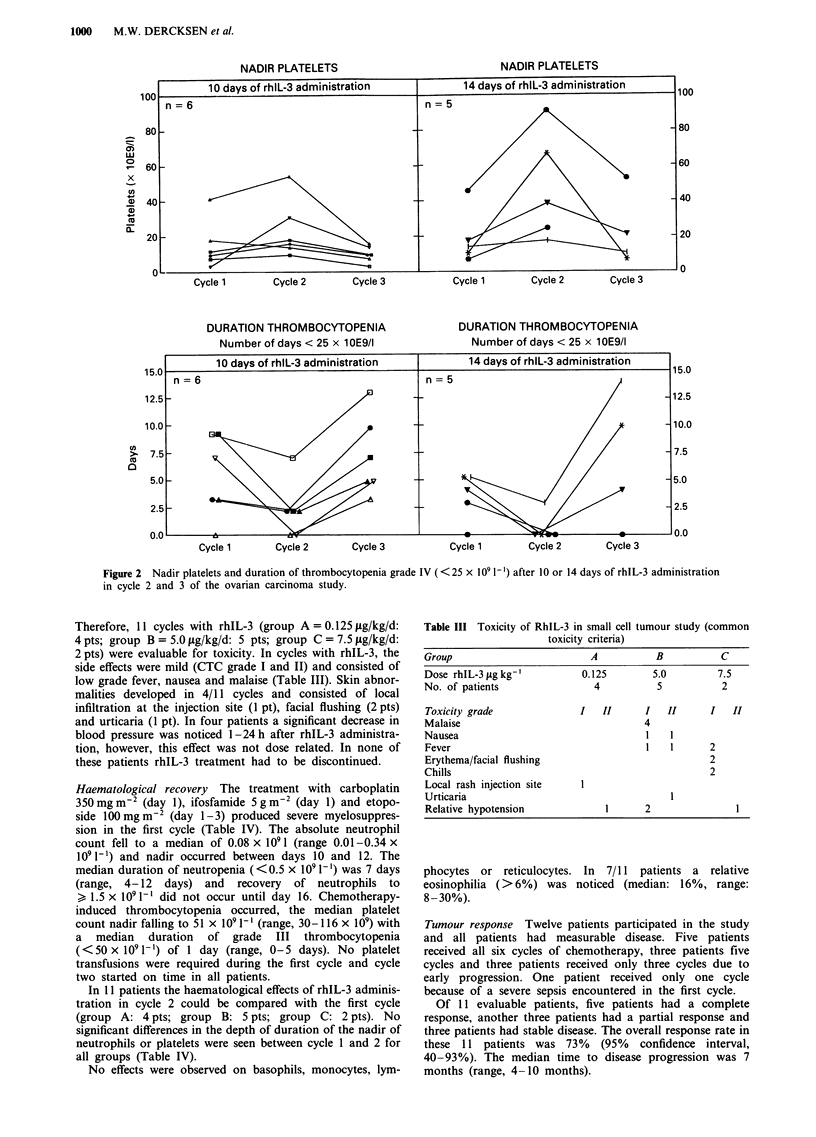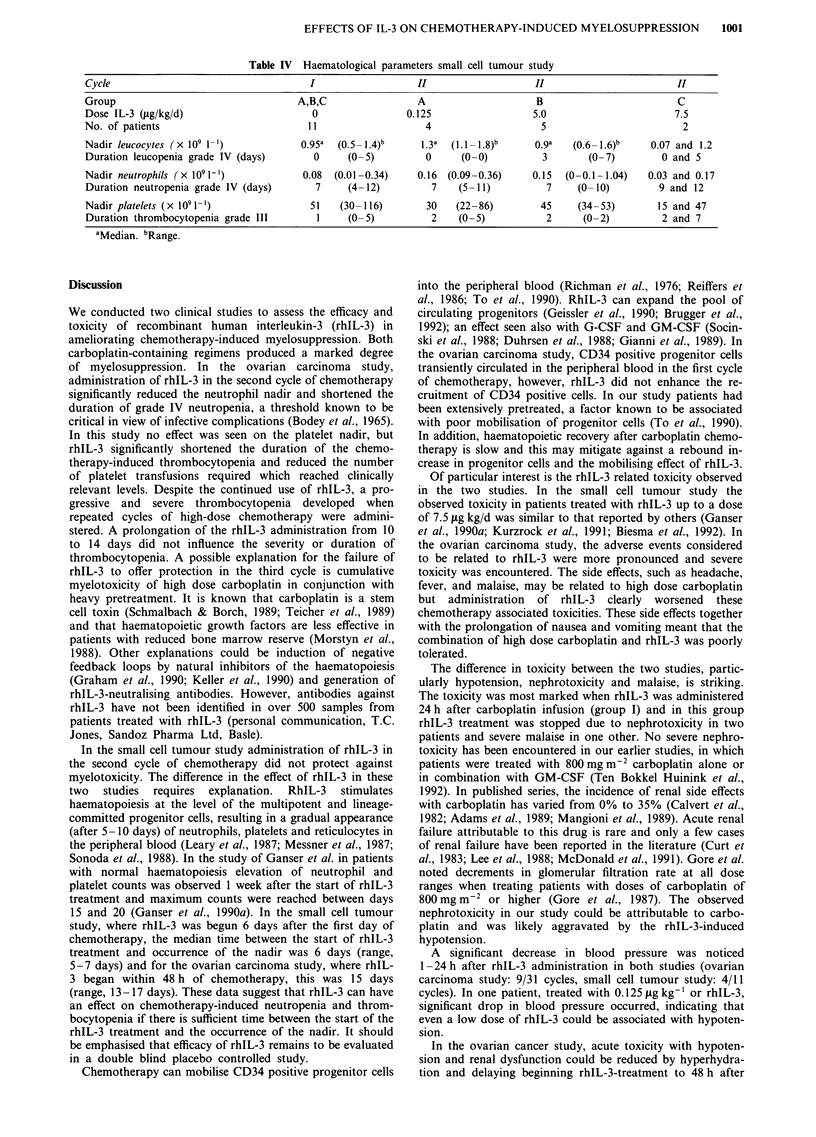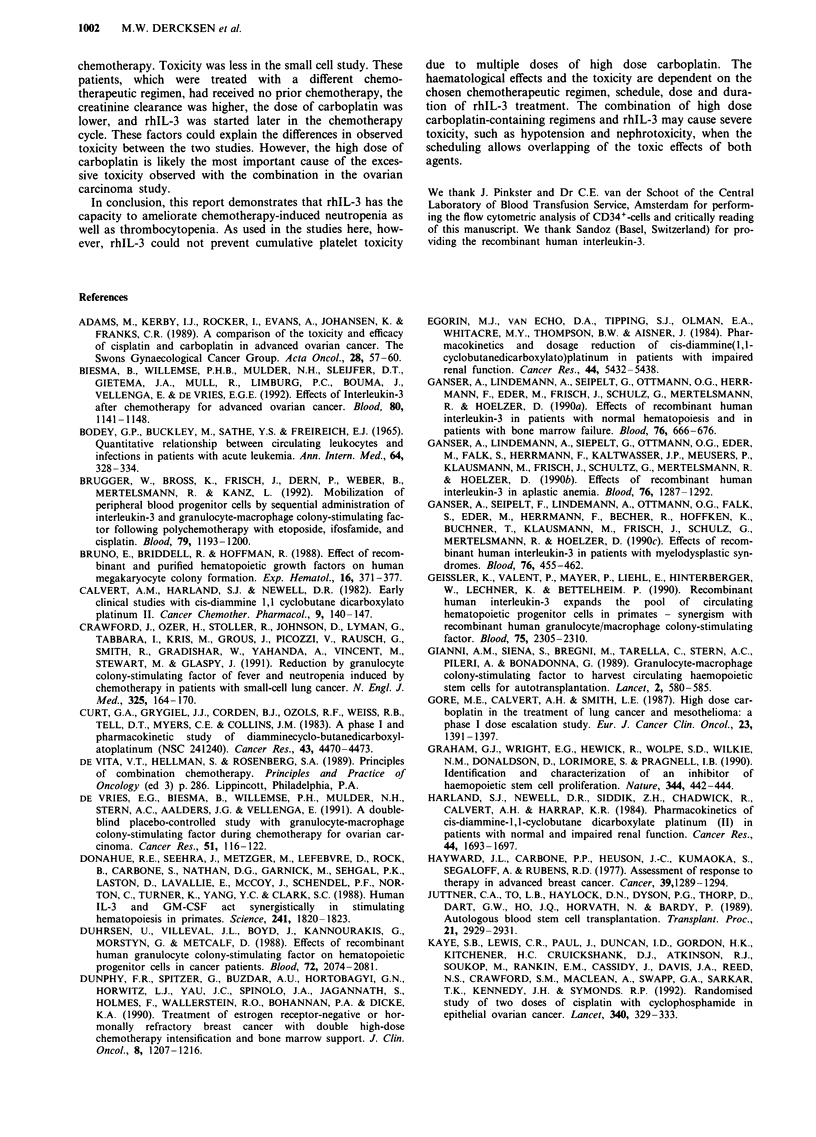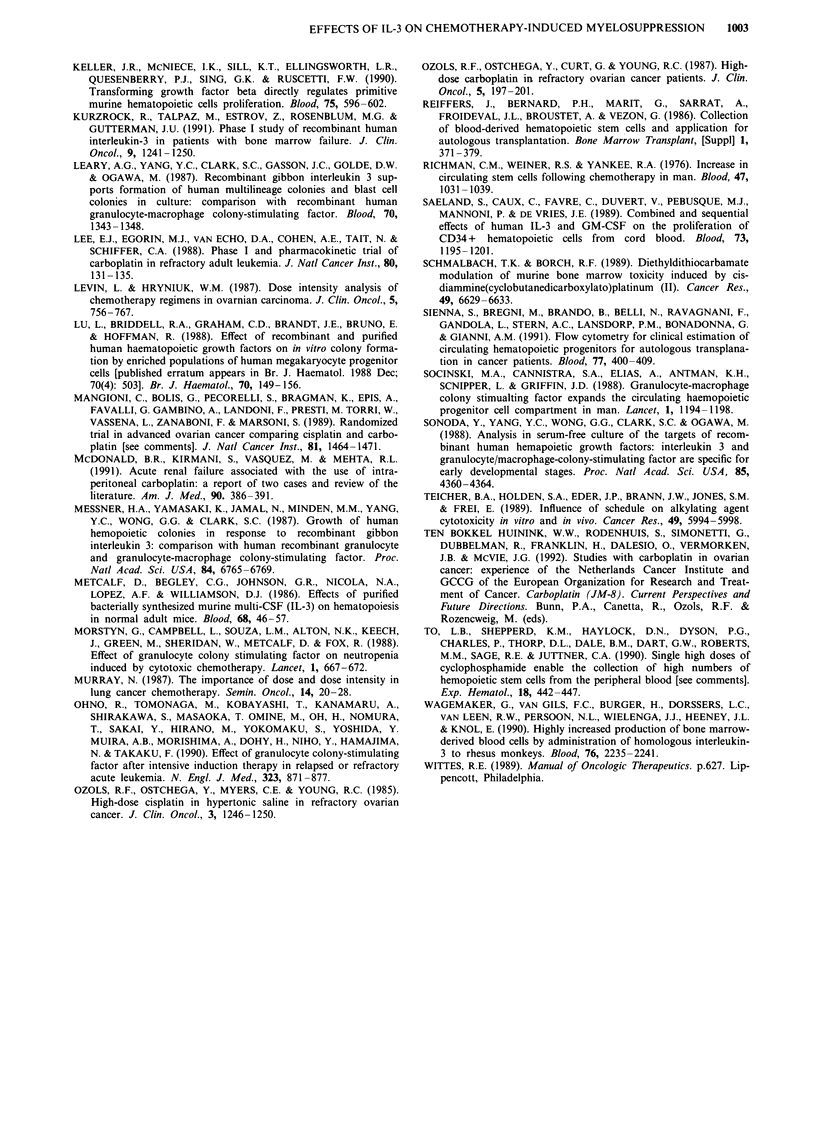# Effects of interleukin-3 on myelosuppression induced by chemotherapy for ovarian cancer and small cell undifferentiated tumours.

**DOI:** 10.1038/bjc.1993.468

**Published:** 1993-11

**Authors:** M. W. Dercksen, K. Hoekman, W. W. ten Bokkel Huinink, E. M. Rankin, R. Dubbelman, H. van Tinteren, J. Wagstaff, H. M. Pinedo

**Affiliations:** Department of Medical Oncology, The Netherlands Cancer Institute/Antoni van Leeuwenhoekhuis, Amsterdam.

## Abstract

Two clinical studies were undertaken to study the toxicity profile and effects of interleukin-3 (rhIL-3) on chemotherapy-induced myelosuppression. Fifteen patients with recurrent ovarian carcinoma were treated with high dose carboplatin (800 mg m-2). All patients received 5.0 micrograms/kg/d rhIL-3 subcutaneously but timing and duration of rhIL-3 treatment differed. Constitutional symptoms were the major toxicity and in addition to the carboplatin-induced nausea and vomiting the combination was poorly tolerated. In 5/15 patients receiving high dose carboplatin rhIL-3 administration was discontinued due to nephrotoxicity (2 x), hypotension, severe malaise and bone pain. In this study, rhIL-3 ameliorated chemotherapy-induced neutropenia as well as thrombocytopenia and reduced the requirement for platelet transfusions in the second cycle of chemotherapy. However, rhIL-3 failed to prevent cumulative platelet toxicity. In the second study 12 patients with small cell undifferentiated cancers were treated with carboplatin, etoposide and ifosfamide. Three dose levels of rhIL-3 were explored (0.125, 5.0 and 7.5 micrograms/kg/d). In this study, toxicity of the treatment was mild, however, no beneficial haematologic effects of rhIL-3 could be demonstrated. In conclusion, the haematological effects of rhIL-3 were modest and dependent on the chemotherapeutic regimen, timing and duration of rhIL-3 treatment (in relation to the expected nadir). In general rhIL-3-induced toxicity was mild, but combination with high dose carboplatin could be hazardous if rhIL-3 is initiated at 24 h after the cytostatic agent.


Br. J. Cancer (1993), 68, 996–1003 © Macmillan Press Ltd., 1993

### Effects of interleukin-3 on myelosuppression induced by chemotherapy for ovarian cancer and small cell undifferentiated tumours

M.W. Dercksen[1,2], K. Hoekman[2], W.W. ten Bokkel Huinink[1], E.M. Rankin[1], R. Dubbelman[1], H. van Tinteren[1], J. Wagstaff[2] & H.M. Pinedo[1,2]

[1]*Department of Medical Oncology, The Netherlands Cancer Institute/Antoni van Leeuwenhoekhuis, Amsterdam;* [2]*Department of Medical Oncology, Free University Hospital, Amsterdam, The Netherlands.*

**Summary** Two clinical studies were undertaken to study the toxicity profile and effects of interleukin-3 (rhIL-3) on chemotherapy-induced myelosuppression.

Fifteen patients with recurrent ovarian carcinoma were treated with high dose carboplatin (800 mg $m^{-2}$). All patients received 5.0 µg/kg/d rhIL-3 subcutaneously but timing and duration of rhIL-3 treatment differed. Constitutional symptoms were the major toxicity and in addition to the carboplatin-induced nausea and vomiting the combination was poorly tolerated. In 5/15 patients receiving high dose carboplatin rhIL-3 administration was discontinued due to nephrotoxicity (2 ×), hypotension, severe malaise and bone pain. In this study, rhIL-3 ameliorated chemotherapy-induced neutropenia as well as thrombocytopenia and reduced the requirement for platelet transfusions in the second cycle of chemotherapy. However, rhIL-3 failed to prevent cumulative platelet toxicity.

In the second study 12 patients with small cell undifferentiated cancers were treated with carboplatin, etoposide and ifosfamide. Three dose levels of rhIL-3 were explored (0.125, 5.0 and 7.5 µg/kg/d). In this study, toxicity of the treatment was mild, however, no beneficial haematologic effects of rhIL-3 could be demonstrated.

In conclusion, the haematological effects of rhIL-3 were modest and dependent on the chemotherapeutic regimen, timing and duration of rhIL-3 treatment (in relation to the expected nadir). In general rhIL-3-induced toxicity was mild, but combination with high dose carboplatin could be hazardous if rhIL-3 is initiated at 24 h after the cytostatic agent.

Increasing the dose of certain cytostatic drugs may improve treatment results in some chemosensitive tumours (Juttner *et al.*, 1989; Dunphy *et al.*, 1990; de Vita *et al.*, 1989). In patients with ovarian cancer, cisplatin and carboplatin demonstrated dose-related activity (Ozols *et al.*, 1985; 1987) and in these patients both probability of response and survival are directly related to dose intensity of these compounds (Levin & Hryniuk, 1987; Kaye *et al.*, 1992). In small cell lung cancer (SCLC) the clinical outcome may also correlate with dose-intensity (Murray, 1987). Thus, increasing dose intensity may improve the efficacy of chemotherapy in the treatment of ovarian cancer and SCLC. Unfortunately, the degree of dose escalation that may be achieved in practice is usually limited by toxicity. For carboplatin the dose limiting toxicity is myelosuppression, predominantly thrombocytopenia (Curt *et al.*, 1983; Egorin *et al.*, 1984; Harland *et al.*, 1984).

Shortening the duration and depth of chemotherapy-induced neutropenia and thrombocytopenia should reduce the incidence and severity of infections and bleeding complications. Use of haematopoietic growth factors may prove beneficial by reducing toxicity of chemotherapy. Granulocyte colony-stimulating factor (G-CSF) and granulocyte-macrophage CSF (GM-CSF) have clearly reduced the depth and extent of neutrophil nadirs, and reduced the incidence of serious infection (Ohno *et al.*, 1990; Crawford *et al.*, 1991; de Vries *et al.*, 1991). Unfortunately, neither G-CSF nor GM-CSF could adequately ameliorate thrombocytopenia due to cytotoxic therapy and therefore may not permit significant dose escalation of drugs which are associated with thrombocytopenia.

Recombinant human interleukin-3 (rhIL-3) is a multilineage haemopoietin that promotes the growth and differentiation of multipotent progenitor cells and committed progenitor cells of the granulocyte/macrophage, eosinophil, basophil, megakaryocyte, and erythroid lineages (Leary *et al.*, 1987; Saeland *et al.*, 1989; Sonoda *et al.*, 1988). Interleukin-3 has been shown to be a potent inducer of megakaryocyte development and thrombopoiesis *in vitro* (Bruno *et al.*, 1988; Lu *et al.*, 1988). Preclinical studies of rhIL-3 in animal models showed a modest and delayed increase in neutrophils, basophils, and eosinophils. Increases in reticulocyte counts and variable increases in platelet counts were also observed (Metcalf *et al.*, 1986; Donahue *et al.*, 1988; Wagemaker *et al.*, 1990).

Clinical experience with rhIL-3 is limited. Several studies with rhIL-3 have been reported, including studies in patients with advanced malignancies (Ganser *et al.*, 1990*a*; Kurzrock *et al.*, 1991), myelodysplastic syndromes (Ganser *et al.*, 1990*c*), and aplastic anaemia (Ganser *et al.*, 1990*b*). Only a few studies have reported rhIL-3 administration in patients with chemotherapy-induced myelosuppression (Kurzrock *et al.*, 1991; Biesma *et al.*, 1992). These studies indicated that rhIL-3 induced a multilineage response showing beneficial effects on platelet counts at various doses.

Before haematophoietic growth factors can achieve widespread use, it is essential to demonstrate their efficacy and safety when given in combination with different chemotherapeutic regimens and in different schedules of administration. We therefore conducted two phase I/II studies with rhIL-3 in patients with ovarian cancer and in patients with small cell undifferentiated tumours. In the former study the bone marrow was challenged with high doses of the myelosuppressive agent carboplatin, while a more conventional regimen was used in the second study. In this paper we present the clinical results of these studies.

### Patients and methods

### *Patient selection*

Patients were treated with chemotherapy plus rhIL-3 in two different protocols. Patients with ovarian carcinoma recurrent after previous platinum-based chemotherapy were included in the first study and patients with small cell undifferentiated tumours in the second study. Most of the small cell tumours originated in the lung and both patients with limited or extensive disease were entered into the study. All patients

Correspondence: H.M. Pinedo, Department of Medical Oncology, The Netherlands Cancer Institute/Antoni van Leeuwenhoekhuis, Plesmanlaan 121, 1066 CX Amsterdam, The Netherlands.
Received 20 April 1993; and in revised form 30 June 1993.

were less than 70 years of age, had a performance status of >60% (Karnofsky scale) and a life expectancy of >2 months. Patients with severe heart, lung, liver (serum bilirubin >25 $\mu$mol $l^{-1}$, SGOT of SGPT >1.25 or normal) or kidney impairment (creatinine clearance ⩽ 50 ml $min^{-1}$) were excluded, as were patients with active infection, evidence of central nervous system metastases or of other malignancies. Evidence of adequate bone marrow function (leucocyte count ⩾ $3.5 \times 10^9 l^{-1}$ and platelet count ⩾ $100 \times 10^9 l^{-1}$) was required. All patients gave informed consent and the protocols were approved by the Ethical and Scientific review committees of the Netherlands Cancer Institute and the Free University Hospital.

*Recombinant human Interleukin-3*

Escherichia Coli derived nonglycosylated rhIL-3 ($2-10 \times 10^6$ U $mg^{-1}$ protein) was provided by Sandoz (Basel, Switzerland) as a lyophilised powder in vials of 150 $\mu$g and 300 $\mu$g. The drug was reconstituted with 1 ml sterile water prior to administration as a daily subcutaneous injection.

*Study design and treatment*

*(a) The ovarian carcinoma study* Fifteen consecutive patients with histologically verified recurrent ovarian carcinoma were entered into this study and treated with 800 mg $m^{-2}$ carboplatin every 4 weeks for a total of four cycles. Carboplatin (Bristol-Myers Squibb, Princeton, NJ, USA) dissolved in 500 ml 5% glucose, was infused over 60 min. Anti-emetic treatment consisted of ondansetron with metoclopramide. RhIL-3 was administered subcutaneously at 5.0 $\mu$g/kg/d from the second cycle onwards and effects of rhIL-3 were compared with the first cycle (control cycle). The patients were divided into three groups of five patients each. Group I was treated with rhIL-3 for 10 days, starting 24 h after carboplatin administration. Group II also received rhIL-3 for 10 days, but starting 48 h after carboplatin infusion. In group III rhIL-3 was administered for 14 days, starting 48 h after chemotherapy. Three patients received rhIL-3 in the first cycle and therefore these cycles were not considered as control cycles.

*(b) The small cell undifferentiated tumour study* Patients with small cell undifferentiated tumours were treated with carboplatin 350 mg $m^{-2}$ (Bristol-Myers Squibb) in a 30 min infusion in 500 ml 5% glucose on day 1, etoposide 100 mg $m^{-2}$ (Bristol-Myers Squibb) as an infusion in 0.9% saline over 30 min on days 1–3 and ifosfamide 5 g $m^{-2}$ (Asta Medica, Frankfurt, Germany) with an equivalent dose of mesna (Asta Medica) in a 24 h infusion on day 1 in 2 litre 0.9% NaCl. This treatment was repeated at 4 weekly intervals for six cycles provided that the disease did not progress.

The first cycle of chemotherapy was given without growth factor. In the second cycle rhIL-3 was administered from days 5–14. The same dose of chemotherapy was given in cycle 1 and 2. Three groups of patients were treated with rhIL-3 at the following dose levels: 0.125, 5.0 and 7.5 $\mu$g/kg/d. The study was terminated because rhIL-3 was no longer supplied.

In both studies red blood cell transfusions were given when the haemoglobin level dropped below 6.0 mmol $l^{-1}$, and platelet transfusions were administered at platelet counts $<10 \times 10^9 l^{-1}$ or when bleeding occurred. No prophylactic antibiotics were administered. Acetaminophen (max. 3 g/day) was given when headache occurred.

*Clinical and laboratory monitoring*

Complete haematological blood counts, including differential cell counts, were performed every 2–3 days. Biochemical analysis was carried out on a weekly basis and creatinine clearance was measured every cycle. Axillary temperature and subjective toxicity were recorded twice daily. Tumour response was evaluated after each cycle of chemotherapy, according to UICC criteria (Hayward *et al.*, 1977).

Toxicity was graded according to the Common Toxicity Criteria (CTC) (Wittes, 1989).

*Flow cytometry*

Circulating haematopoietic progenitor cells were identified in peripheral blood obtained from patients recovering from high dose carboplatin using flow cytometry as described by Siena *et al.* (1991). Cells expressing the surface membrane CD34 antigen were identified with the FITC-conjugated CD34 murine monoclonal antibody (8G12 kindly provided by Dr Peter Lansdorp, Vancouver, BC, Canada). The percentage of $CD34^+$-cells were determined by flow cytometric analysis using a FACScan (Becton and Dickinson, Saolo Paolo, CA, USA).

*Statistical analysis*

Differences between the subgroups with regard to factors that might influence haematopoietic recovery were tested with the Fisher exact test and Wilcoxon rank test. The Wilcoxon matched-pairs signed rank test was used to test differences in haematological values between cycles.

### Results

*Ovarian carcinoma study*

*Patient characteristics* Fifteen patients were entered on study. The median age was 53 years (range, 34–69 years) and the median performance status was 80% (range, 60–100%) (Karnofsky scale). Five patients had a late relapse (>1 year), three patients had an early relapse (<1 year) following first-line chemotherapy and seven patients were refractory to previous chemotherapy. All patients had received at least six cycles of platinum-based chemotherapy (median 6, range: 6–15 cycles). The median time from diagnosis was 28 months (range, 6–154 months) and from last chemotherapy 6 months (range 1–64 months). Creatinine clearance (mean ± s.d.) at entry was 75 ± 8.5 ml $min^{-1}$, 80 ± 10.7 ml $min^{-1}$, and 83 ± 17.3 ml $min^{-1}$ in group I, II and III, respectively. No significant differences could be found between the three patient populations, particularly when factors that might influence haematopoietic recovery were considered, i.e. performance status, age and time since or extent of previous pretreatment.

*Side effects* RhIL-3 was administered in 31 cycles of carboplatin which were evaluable for toxicity (group I: 7, group II: 12, group III: 12). In these cycles, the most frequently observed rhIL-3-related side effects were low-grade fever (CTC grade I) and headache (CTC grade I–II) (Table I), which usually responded to acetaminophen. Facial flushing and diffuse erythema were noticed in a majority of the cycles (19/31). These usually started 1–6 h after rhIL-3 administration and were most pronounced during the first days of treatment. In two patients local infiltrates at the injection site were seen. Other minor side effects consisted of muscle or bone pain and chills.

Beside the myelosuppression, side effects observed in cycles without rhIL-3 (control cycles) consisted of malaise, headache and chemotherapy-related nausea and vomiting (CTC grade III and IV), which had stopped within a week of chemotherapy. In cycles with rhIL-3, nausea and vomiting were more pronounced and extended over a longer period of time. After day 7, patients with rhIL-3 still experienced episodes of nausea (14/31) usually accompanied by vomiting (11/31) (Table I). Together with the malaise, headache and fever, this meant that high dose carboplatin in combination with rhIL-3 was poorly tolerated.

In 5/15 patients rhIL-3 was stopped due to toxicity. This occurred three times in group I in the first cycle with rhIL-3

**Table I** Adverse events ovarian carcinoma study (common toxicity criteria)

| | *Cycles without rhIL-3 (n = 12)* | | *Cycles with rhIL-3 (n = 31)* | | | |
|---|---|---|---|---|---|---|
| *Toxicity grade* | *I* | *II* | *I* | *II* | *III* | *IV* |
| Nausea (days 7–16) | 3 | | 8 | 6 | | |
| Vomiting (days 7–16) | 2 | | 7 | 4 | | |
| Malaise | 10 | 2 | 22 | 7 | 2 (1[a]) | |
| Headache | 3 | | 9 | 7 | | |
| Bone pain | | | 5 | | 1[a] | |
| Muscle pain | | | 6 | | | |
| Chills | 1 | | 8 | 3 | | |
| Fever | 3 | 3 | 15 | 16 | | |
| Erythema/facial flushing | 1 | | 19 | | | |
| Local rash injection site | | | 3 | | | |
| Diarrhea | | 1 | 3 | 3 | | |
| Elevation serum creatinine | | | 2 | | 1[a] | 1[a] |
| Relative hypotension | | | 5 | 1 | 3 (1[a]) | |

[a]Main reason for stopping rhIL-3 administration.

and twice in groups II and III in the third cycle. The reasons for discontinuation were nephrotoxicity in two patients and severe malaise, hypotension or bone pain in three other patients. Renal function impairment and a drop in blood pressure occurred in 2/31 and 5/31 cycles, respectively.

In group I, when rhIL-3 was administered 24 h after carboplatin infusion, the toxicity was most marked. One to 24 h after rhIL-3 administration all five patients developed a drop in mean arterial pressure ranging from 20% to 45% of initial values and in two of these patients serum creatinine levels were found to be elevated (360 and 365 $\mu$mol $l^{-1}$) on days 4 and 5. Both patients experienced grade IV nausea and vomiting, high grade fever (>39°C) and facial flushing and became dehydrated. After discontinuation of rhIL-3, blood pressure normalised in both patients. In one patient serum creatinine levels returned to normal in 1 week. In the other patient, with an initial marginal renal function, serum creatinine levels rose to 713 $\mu$mol $l^{-1}$ despite appropriate supportive measures. Two weeks later she developed septicaemia with severe thrombocytopenia. At the request of the patient treatment was discontinued and she died from haemorrhagic complications.

In group II, rhIL-3 administration was started 48 h instead of 24 h after carboplatin infusion. In view of the observed toxicity in group I, these patients were extensively hydrated pre- and post-carboplatin administration. In the remaining ten patients no further nephrotoxicity was observed. Creatinine clearance at entry to each cycle did not differ significantly among the three groups (Table II). One patient experienced a 40% decrease in mean arterial blood pressure 12 h after the first rhIL-3 administration in the fourth cycle and subsequently rhIL-3 treated was stopped. Forty-eight hours after rhIL-3 administration blood pressure had returned to normal. No changes in blood pressure requiring therapy were observed in the other nine patients.

*Haematological recovery* A total of 27 cycles of rhIL-3 were completed in 12 patients. Eleven patients were evaluable for efficacy of rhIL-3, one patient was not evaluable because of dose reduction of carboplatin in cycle 2. Treatment with 800 mg $m^{-2}$ carboplatin in cycle 1 induced severe myelosuppression in all patients (Table II). The absolute neutrophil count fell to a median of $0.15 \times 10^9 l^{-1}$ and the nadir occurred between days 16 and 21. Recovery of neutrophils (to $\geqslant 1.5 \times 10^9 l^{-1}$) did not occur until day 26.

No significant difference in neutrophil or platelet nadir or in duration of neutropenia and thrombocytopenia was observed between 10 days (group II) and 14 days (group III) of rhIL-3 administration. The data from these two groups have therefore been combined in Table II. Administration of rhIL-3 in the second cycle of chemotherapy significantly reduced both the neutrophil nadir and the duration of neutropenia. The median absolute neutrophil count nadir increased to $0.27 \times 10^9 l^{-1}$ ($P<0.05$) and duration of grade IV neutropenia ($\leqslant 0.5 \times 10^9 l^{-1}$) was shortened to a median of 3 days from a median of 9 days in cycle 1 ($P<0.01$) (Figure 1 and Table II). No significant difference was found for the absolute neutrophil count on day 28 of cycle 2 compared with the same time in cycle 1. However, postponement of chemotherapy by 1 or 2 weeks because of leucopenia ($<3.5 \times 10^9 l^{-1}$) occurred in 4/10 patients after the second cycle (with rhIL-3), as compared with postponement after the first cycle in 8/11 patients (without rhIL-3).

The platelet nadirs were not significantly different in cycles

**Table II** Haematological parameters in the ovarian carcinoma study

| *Cycle* | *1* | | *2* | | *3* | |
|---|---|---|---|---|---|---|
| Dose IL-3 (μg/kg/d) | 0 | | 5.0 | | 5.0 | |
| No. of patients | 11 | | 11 | | 10 | |
| Mean creatinine clearance at entry of cycle (ml $min^{-1}$) | 78.7 | | 75.1 | | 78.6 | |
| Nadir *leucocytes* ($\times 10^9 l^{-1}$) | 1.0[a] | (0.4–1.9)[b] | 1.3[a] | (0.6–2.1)[b] | 1.2[a] | (1.0–2.1)[b] |
| Duration leucopenia grade IV (days) | 0 | (0–14) | 0 | (0–3) | 0 | (0–0) |
| Nadir *neutrophils* ($\times 10^9 l^{-1}$) | 0.15 | (0–0.90) | 0.27 | (0.05–0.85) | 0.34 | (0–0.98) |
| Duration neutropenia grade IV (days) | 9 | (0–16) | 3 | (0–9) | 4 | (0–14) |
| Nadir *platelets* ($\times 10^9 l^{-1}$) | 15 | (3–45) | 18 | (9–91) | 9 | (3–20) |
| Duration thrombocytopenia grade III (days) | 9 | (0–14) | 5 | (0–9) | 13.5 | (3–20) |
| Duration thrombocytopenia grade IV (days) | 4 | (0–9) | 2 | (0–7) | 6 | (3–14) |
| Total no. platelet transfusions | 9 | | 2 | | 16 | |
| Pts. requiring platelet transfusion (%) | 7 | (64%) | 1 | (9%) | 6 | (60%) |
| Postponement of next cycle (%) | 8/11 | (73%) | 4/10 | (40%) | 4/5 | (80%) |

[a]Median. [b]Range.

**Figure 1** Nadir of neutrophils and duration of neutropenia grade IV ($<0.5 \times 10^9\,l^{-1}$) after 10 or 14 days of rhIL-3 administration in cycle 2 and 3 of the ovarian carcinoma study.

1 and 2 (Figure 2 and Table II). However, the median duration of thrombocytopenia grade III and IV was significantly shorter (cycle 1: grade III: 9 days; grade IV: 4 days *vs* cycle 2: grade III: 5 days ($P<0.05$); grade IV: 2 days ($P<0.01$)). In addition, the recovery of platelets was faster and platelet numbers were higher on day 21 of cycle 2 than of cycle 1 ($P<0.005$). Platelet transfusion were necessary in 1/11 patients (9%) in the second cycle of chemotherapy compared with 7/11 patients (64%) in the first cycle ($P<0.05$).

RhIL-3 also demonstrated an effect on the eosinophil count. In seven of the 12 patients who completed rhIL-3 administration, a relative eosinophilia (>6%) was noticed (day 12, median: 11%, range: 7–34%) compared with 2/12 patients in control cycles (range: 7–8%). No effects were observed on basophils, monocytes, lymphocytes or myeloid progenitor cells. At day 14 and 21 median reticulocyte counts were higher in cycle 2 than in the first cycle (day 14: 7% *vs* 0%; day 21: 21% *vs* 4%).

In eight patients CD34+ cells were measured in peripheral blood samples. Peak levels in cycle 1 and 2 were not significantly different (median percentage CD34+ cells in mononuclear cell fraction in cycle 1 without rhIL-3: 0.55% (range 0–2.8); with rhIL-3: 0.3% (range 0–1.8)). In fact in five of the eight patients the percentage CD34+ cells was higher in cycle 1.

Administration of rhIL-3 in ten patients in the third cycle of chemotherapy again demonstrated an effect on the neutrophil nadir and on the duration of neutropenia as compared with the first chemotherapy cycle. The median nadir of platelet count was however significantly lower than in the first cycle ($P<0.05$) and the duration of thrombocytopenia grade III and IV was longer ($P<0.01$ and $P<0.05$) (Table II). Platelet transfusions were necessary in six of the ten patients (60%) and in two patients chemotherapy was stopped because of a prolonged thrombocytopenia.

*Tumour response* Ten of 14 patients who were evaluable for response, showed a response to treatment (two clinical complete responses, eight partial responses, one stable disease and three progressive disease). The overall response rate in these 14 patients was 71% (95% confidence interval, 41–90%). The median time to disease progression was 8 months (range, 2–11+ months).

### *Small cell undifferentiated tumour study*

*Patient characteristics* Twelve patients with histologically proven small cell undifferentiated tumours were entered in this study. The median age was 64 years (range, 35–70 years) and the median performance status was 80% (range, 70–100%) (Karnofsky scale). Nine patients had small cell cancers of the lung (five patients had limited and four extended disease). The other patients had a small cell undifferentiated tumour originating from hypopharynx, cervix or thyroid respectively. Five patients were entered in group A (rhIL-3: 0.125 µg/kg/d), five patients in group B (5.0 µg/kg/d) and two patients were entered in group C (7.5 µg/kg/d). Creatinine clearance (mean ± s.d.) at entry was 112 ± 19.8 ml min$^{-1}$, 121 ± 34.4 ml min$^{-1}$, and 104 ± 5.0 ml min$^{-1}$ in group A, B and C, respectively. There was no significant difference between the three patient groups in terms of performance status or age.

*Side effects* In the cycles without rhIL-3 toxicity consisted of chemotherapy-induced nausea and vomiting (CTC grade III). One patient in group I encountered a severe sepsis in the first cycle and was subsequently removed from the study.

**Figure 2** Nadir platelets and duration of thrombocytopenia grade IV ($<25 \times 10^9\,l^{-1}$) after 10 or 14 days of rhIL-3 administration in cycle 2 and 3 of the ovarian carcinoma study.

Therefore, 11 cycles with rhIL-3 (group A = 0.125 μg/kg/d: 4 pts; group B = 5.0 μg/kg/d: 5 pts; group C = 7.5 μg/kg/d: 2 pts) were evaluable for toxicity. In cycles with rhIL-3, the side effects were mild (CTC grade I and II) and consisted of low grade fever, nausea and malaise (Table III). Skin abnormalities developed in 4/11 cycles and consisted of local infiltration at the injection site (1 pt), facial flushing (2 pts) and urticaria (1 pt). In four patients a significant decrease in blood pressure was noticed 1–24 h after rhIL-3 administration, however, this effect was not dose related. In none of these patients rhIL-3 treatment had to be discontinued.

*Haematological recovery* The treatment with carboplatin 350 mg $m^{-2}$ (day 1), ifosfamide 5 g $m^{-2}$ (day 1) and etoposide 100 mg $m^{-2}$ (day 1–3) produced severe myelosuppression in the first cycle (Table IV). The absolute neutrophil count fell to a median of $0.08 \times 10^9$ l (range $0.01-0.34 \times 10^9\,l^{-1}$) and nadir occurred between days 10 and 12. The median duration of neutropenia ($<0.5 \times 10^9\,l^{-1}$) was 7 days (range, 4–12 days) and recovery of neutrophils to $\geqslant 1.5 \times 10^9\,l^{-1}$ did not occur until day 16. Chemotherapy-induced thrombocytopenia occurred, the median platelet count nadir falling to $51 \times 10^9\,l^{-1}$ (range, $30-116 \times 10^9$) with a median duration of grade III thrombocytopenia ($<50 \times 10^9\,l^{-1}$) of 1 day (range, 0–5 days). No platelet transfusions were required during the first cycle and cycle two started on time in all patients.

In 11 patients the haematological effects of rhIL-3 administration in cycle 2 could be compared with the first cycle (group A: 4 pts; group B: 5 pts; group C: 2 pts). No significant differences in the depth of duration of the nadir of neutrophils or platelets were seen between cycle 1 and 2 for all groups (Table IV).

No effects were observed on basophils, monocytes, lymphocytes or reticulocytes. In 7/11 patients a relative eosinophilia (>6%) was noticed (median: 16%, range: 8–30%).

**Table III** Toxicity of RhIL-3 in small cell tumour study (common toxicity criteria)

| *Group* | *A* | | *B* | | *C* | |
|---|---|---|---|---|---|---|
| Dose rhIL-3 μg $kg^{-1}$ | 0.125 | | 5.0 | | 7.5 | |
| No. of patients | 4 | | 5 | | 2 | |
| *Toxicity grade* | *I* | *II* | *I* | *II* | *I* | *II* |
| Malaise | | | 4 | | | |
| Nausea | | | 1 | 1 | | |
| Fever | | | 1 | 1 | 2 | |
| Erythema/facial flushing | | | | | 2 | |
| Chills | | | | | 2 | |
| Local rash injection site | 1 | | | | | |
| Urticaria | | | | 1 | | |
| Relative hypotension | | 1 | 2 | | | 1 |

*Tumour response* Twelve patients participated in the study and all patients had measurable disease. Five patients received all six cycles of chemotherapy, three patients five cycles and three patients received only three cycles due to early progression. One patient received only one cycle because of a severe sepsis encountered in the first cycle.

Of 11 evaluable patients, five patients had a complete response, another three patients had a partial response and three patients had stable disease. The overall response rate in these 11 patients was 73% (95% confidence interval, 40–93%). The median time to disease progression was 7 months (range, 4–10 months).

**Table IV** Haematological parameters small cell tumour study

| *Cycle* | *I* | | *II* | | *II* | | *II* |
|---|---|---|---|---|---|---|---|
| Group | A,B,C | | A | | B | | C |
| Dose IL-3 (μg/kg/d) | 0 | | 0.125 | | 5.0 | | 7.5 |
| No. of patients | 11 | | 4 | | 5 | | 2 |
| Nadir *leucocytes* ( $\times 10^9 l^{-1}$) | 0.95[a] | (0.5–1.4)[b] | 1.3[a] | (1.1–1.8)[b] | 0.9[a] | (0.6–1.6)[b] | 0.07 and 1.2 |
| Duration leucopenia grade IV (days) | 0 | (0–5) | 0 | (0–0) | 3 | (0–7) | 0 and 5 |
| Nadir *neutrophils* ( $\times 10^9 l^{-1}$) | 0.08 | (0.01–0.34) | 0.16 | (0.09–0.36) | 0.15 | (0–0.1–1.04) | 0.03 and 0.17 |
| Duration neutropenia grade IV (days) | 7 | (4–12) | 7 | (5–11) | 7 | (0–10) | 9 and 12 |
| Nadir *platelets* ( $\times 10^9 l^{-1}$) | 51 | (30–116) | 30 | (22–86) | 45 | (34–53) | 15 and 47 |
| Duration thrombocytopenia grade III | 1 | (0–5) | 2 | (0–5) | 2 | (0–2) | 2 and 7 |

[a]Median. [b]Range.

### Discussion

We conducted two clinical studies to assess the efficacy and toxicity of recombinant human interleukin-3 (rhIL-3) in ameliorating chemotherapy-induced myelosuppression. Both carboplatin-containing regimens produced a marked degree of myelosuppression. In the ovarian carcinoma study, administration of rhIL-3 in the second cycle of chemotherapy significantly reduced the neutrophil nadir and shortened the duration of grade IV neutropenia, a threshold known to be critical in view of infective complications (Bodey *et al.*, 1965). In this study no effect was seen on the platelet nadir, but rhIL-3 significantly shortened the duration of the chemotherapy-induced thrombocytopenia and reduced the number of platelet transfusions required which reached clinically relevant levels. Despite the continued use of rhIL-3, a progressive and severe thrombocytopenia developed when repeated cycles of high-dose chemotherapy were administered. A prolongation of the rhIL-3 administration from 10 to 14 days did not influence the severity or duration of thrombocytopenia. A possible explanation for the failure of rhIL-3 to offer protection in the third cycle is cumulative myelotoxicity of high dose carboplatin in conjunction with heavy pretreatment. It is known that carboplatin is a stem cell toxin (Schmalbach & Borch, 1989; Teicher *et al.*, 1989) and that haematopoietic growth factors are less effective in patients with reduced bone marrow reserve (Morstyn *et al.*, 1988). Other explanations could be induction of negative feedback loops by natural inhibitors of the haematopoiesis (Graham *et al.*, 1990; Keller *et al.*, 1990) and generation of rhIL-3-neutralising antibodies. However, antibodies against rhIL-3 have not been identified in over 500 samples from patients treated with rhIL-3 (personal communication, T.C. Jones, Sandoz Pharma Ltd, Basle).

In the small cell tumour study administration of rhIL-3 in the second cycle of chemotherapy did not protect against myelotoxicity. The difference in the effect of rhIL-3 in these two studies requires explanation. RhIL-3 stimulates haematopoiesis at the level of the multipotent and lineage-committed progenitor cells, resulting in a gradual appearance (after 5–10 days) of neutrophils, platelets and reticulocytes in the peripheral blood (Leary *et al.*, 1987; Messner *et al.*, 1987; Sonoda *et al.*, 1988). In the study of Ganser *et al.* in patients with normal haematopoiesis elevation of neutrophil and platelet counts was observed 1 week after the start of rhIL-3 treatment and maximum counts were reached between days 15 and 20 (Ganser *et al.*, 1990*a*). In the small cell tumour study, where rhIL-3 was begun 6 days after the first day of chemotherapy, the median time between the start of rhIL-3 treatment and occurrence of the nadir was 6 days (range, 5–7 days) and for the ovarian carcinoma study, where rhIL-3 began within 48 h of chemotherapy, this was 15 days (range, 13–17 days). These data suggest that rhIL-3 can have an effect on chemotherapy-induced neutropenia and thrombocytopenia if there is sufficient time between the start of the rhIL-3 treatment and the occurrence of the nadir. It should be emphasised that efficacy of rhIL-3 remains to be evaluated in a double blind placebo controlled study.

Chemotherapy can mobilise CD34 positive progenitor cells into the peripheral blood (Richman *et al.*, 1976; Reiffers *et al.*, 1986; To *et al.*, 1990). RhIL-3 can expand the pool of circulating progenitors (Geissler *et al.*, 1990; Brugger *et al.*, 1992); an effect seen also with G-CSF and GM-CSF (Socinski *et al.*, 1988; Duhrsen *et al.*, 1988; Gianni *et al.*, 1989). In the ovarian carcinoma study, CD34 positive progenitor cells transiently circulated in the peripheral blood in the first cycle of chemotherapy, however, rhIL-3 did not enhance the recruitment of CD34 positive cells. In our study patients had been extensively pretreated, a factor known to be associated with poor mobilisation of progenitor cells (To *et al.*, 1990). In addition, haematopoietic recovery after carboplatin chemotherapy is slow and this may mitigate against a rebound increase in progenitor cells and the mobilising effect of rhIL-3.

Of particular interest is the rhIL-3 related toxicity observed in the two studies. In the small cell tumour study the observed toxicity in patients treated with rhIL-3 up to a dose of 7.5 μg kg/d was similar to that reported by others (Ganser *et al.*, 1990*a*; Kurzrock *et al.*, 1991; Biesma *et al.*, 1992). In the ovarian carcinoma study, the adverse events considered to be related to rhIL-3 were more pronounced and severe toxicity was encountered. The side effects, such as headache, fever, and malaise, may be related to high dose carboplatin but administration of rhIL-3 clearly worsened these chemotherapy associated toxicities. These side effects together with the prolongation of nausea and vomiting meant that the combination of high dose carboplatin and rhIL-3 was poorly tolerated.

The difference in toxicity between the two studies, particularly hypotension, nephrotoxicity and malaise, is striking. The toxicity was most marked when rhIL-3 was administered 24 h after carboplatin infusion (group I) and in this group rhIL-3 treatment was stopped due to nephrotoxicity in two patients and severe malaise in one other. No severe nephrotoxicity has been encountered in our earlier studies, in which patients were treated with 800 mg $m^{-2}$ carboplatin alone or in combination with GM-CSF (Ten Bokkel Huinink *et al.*, 1992). In published series, the incidence of renal side effects with carboplatin has varied from 0% to 35% (Calvert *et al.*, 1982; Adams *et al.*, 1989; Mangioni *et al.*, 1989). Acute renal failure attributable to this drug is rare and only a few cases of renal failure have been reported in the literature (Curt *et al.*, 1983; Lee *et al.*, 1988; McDonald *et al.*, 1991). Gore *et al.* noted decrements in glomerular filtration rate at all dose ranges when treating patients with doses of carboplatin of 800 mg $m^{-2}$ or higher (Gore *et al.*, 1987). The observed nephrotoxicity in our study could be attributable to carboplatin and was likely aggravated by the rhIL-3-induced hypotension.

A significant decrease in blood pressure was noticed 1–24 h after rhIL-3 administration in both studies (ovarian carcinoma study: 9/31 cycles, small cell tumour study: 4/11 cycles). In one patient, treated with 0.125 μg $kg^{-1}$ or rhIL-3, significant drop in blood pressure occurred, indicating that even a low dose of rhIL-3 could be associated with hypotension.

In the ovarian cancer study, acute toxicity with hypotension and renal dysfunction could be reduced by hyperhydration and delaying beginning rhIL-3-treatment to 48 h after chemotherapy. Toxicity was less in the small cell study. These patients, which were treated with a different chemotherapeutic regimen, had received no prior chemotherapy, the creatinine clearance was higher, the dose of carboplatin was lower, and rhIL-3 was started later in the chemotherapy cycle. These factors could explain the differences in observed toxicity between the two studies. However, the high dose of carboplatin is likely the most important cause of the excessive toxicity observed with the combination in the ovarian carcinoma study.

In conclusion, this report demonstrates that rhIL-3 has the capacity to ameliorate chemotherapy-induced neutropenia as well as thrombocytopenia. As used in the studies here, however, rhIL-3 could not prevent cumulative platelet toxicity due to multiple doses of high dose carboplatin. The haematological effects and the toxicity are dependent on the chosen chemotherapeutic regimen, schedule, dose and duration of rhIL-3 treatment. The combination of high dose carboplatin-containing regimens and rhIL-3 may cause severe toxicity, such as hypotension and nephrotoxicity, when the scheduling allows overlapping of the toxic effects of both agents.

We thank J. Pinkster and Dr C.E. van der Schoot of the Central Laboratory of Blood Transfusion Service, Amsterdam for performing the flow cytometric analysis of CD34$^+$-cells and critically reading of this manuscript. We thank Sandoz (Basel, Switzerland) for providing the recombinant human interleukin-3.

### References

ADAMS, M., KERBY, I.J., ROCKER, I., EVANS, A., JOHANSEN, K. & FRANKS, C.R. (1989). A comparison of the toxicity and efficacy of cisplatin and carboplatin in advanced ovarian cancer. The Swons Gynaecological Cancer Group. *Acta Oncol.*, **28**, 57–60.

BIESMA, B., WILLEMSE, P.H.B., MULDER, N.H., SLEIJFER, D.T., GIETEMA, J.A., MULL, R., LIMBURG, P.C., BOUMA, J., VELLENGA, E. & DE VRIES, E.G.E. (1992). Effects of Interleukin-3 after chemotherapy for advanced ovarian cancer. *Blood*, **80**, 1141–1148.

BODEY, G.P., BUCKLEY, M., SATHE, Y.S. & FREIREICH, E.J. (1965). Quantitative relationship between circulating leukocytes and infections in patients with acute leukemia. *Ann. Intern. Med.*, **64**, 328–334.

BRUGGER, W., BROSS, K., FRISCH, J., DERN, P., WEBER, B., MERTELSMANN, R. & KANZ, L. (1992). Mobilization of peripheral blood progenitor cells by sequential administration of interleukin-3 and granulocyte-macrophage colony-stimulating factor following polychemotherapy with etoposide, ifosfamide, and cisplatin. *Blood*, **79**, 1193–1200.

BRUNO, E., BRIDDELL, R. & HOFFMAN, R. (1988). Effect of recombinant and purified hematopoietic growth factors on human megakaryocyte colony formation. *Exp. Hematol.*, **16**, 371–377.

CALVERT, A.M., HARLAND, S.J. & NEWELL, D.R. (1982). Early clinical studies with cis-diammine 1,1 cyclobutane dicarboxylato platinum II. *Cancer Chemother. Pharmacol.*, **9**, 140–147.

CRAWFORD, J., OZER, H., STOLLER, R., JOHNSON, D., LYMAN, G., TABBARA, I., KRIS, M., GROUS, J., PICOZZI, V., RAUSCH, G., SMITH, R., GRADISHAR, W., YAHANDA, A., VINCENT, M., STEWART, M. & GLASPY, J. (1991). Reduction by granulocyte colony-stimulating factor of fever and neutropenia induced by chemotherapy in patients with small-cell lung cancer. *N. Engl. J. Med.*, **325**, 164–170.

CURT, G.A., GRYGIEL, J.J., CORDEN, B.J., OZOLS, R.F., WEISS, R.B., TELL, D.T., MYERS, C.E. & COLLINS, J.M. (1983). A phase I and pharmacokinetic study of diamminecyclo-butanedicarboxylatoplatinum (NSC 241240). *Cancer Res.*, **43**, 4470–4473.

DE VITA, V.T., HELLMAN, S. & ROSENBERG, S.A. (1989). Principles of combination chemotherapy. *Principles and Practice of Oncology* (ed 3) p. 286. Lippincott, Philadelphia, P.A.

DE VRIES, E.G., BIESMA, B., WILLEMSE, P.H., MULDER, N.H., STERN, A.C., AALDERS, J.G. & VELLENGA, E. (1991). A double-blind placebo-controlled study with granulocyte-macrophage colony-stimulating factor during chemotherapy for ovarian carcinoma. *Cancer Res.*, **51**, 116–122.

DONAHUE, R.E., SEEHRA, J., METZGER, M., LEFEBVRE, D., ROCK, B., CARBONE, S., NATHAN, D.G., GARNICK, M., SEHGAL, P.K., LASTON, D., LAVALLIE, E., MCCOY, J., SCHENDEL, P.F., NORTON, C., TURNER, K., YANG, Y.C. & CLARK, S.C. (1988). Human IL-3 and GM-CSF act synergistically in stimulating hematopoiesis in primates. *Science*, **241**, 1820–1823.

DUHRSEN, U., VILLEVAL, J.L., BOYD, J., KANNOURAKIS, G., MORSTYN, G. & METCALF, D. (1988). Effects of recombinant human granulocyte colony-stimulating factor on hematopoietic progenitor cells in cancer patients. *Blood*, **72**, 2074–2081.

DUNPHY, F.R., SPITZER, G., BUZDAR, A.U., HORTOBAGYI, G.N., HORWITZ, L.J., YAU, J.C., SPINOLO, J.A., JAGANNATH, S., HOLMES, F., WALLERSTEIN, R.O., BOHANNAN, P.A. & DICKE, K.A. (1990). Treatment of estrogen receptor-negative or hormonally refractory breast cancer with double high-dose chemotherapy intensification and bone marrow support. *J. Clin. Oncol.*, **8**, 1207–1216.

EGORIN, M.J., VAN ECHO, D.A., TIPPING, S.J., OLMAN, E.A., WHITACRE, M.Y., THOMPSON, B.W. & AISNER, J. (1984). Pharmacokinetics and dosage reduction of cis-diammine(1,1-cyclobutanedicarboxylato)platinum in patients with impaired renal function. *Cancer Res.*, **44**, 5432–5438.

GANSER, A., LINDEMANN, A., SEIPELT, G., OTTMANN, O.G., HERRMANN, F., EDER, M., FRISCH, J., SCHULZ, G., MERTELSMANN, R. & HOELZER, D. (1990*a*). Effects of recombinant human interleukin-3 in patients with normal hematopoiesis and in patients with bone marrow failure. *Blood*, **76**, 666–676.

GANSER, A., LINDEMANN, A., SIEPELT, G., OTTMANN, O.G., EDER, M., FALK, S., HERRMANN, F., KALTWASSER, J.P., MEUSERS, P., KLAUSMANN, M., FRISCH, J., SCHULTZ, G., MERTELSMANN, R. & HOELZER, D. (1990*b*). Effects of recombinant human interleukin-3 in aplastic anemia. *Blood*, **76**, 1287–1292.

GANSER, A., SEIPELT, F., LINDEMANN, A., OTTMANN, O.G., FALK, S., EDER, M., HERRMANN, F., BECHER, R., HOFFKEN, K., BUCHNER, T., KLAUSMANN, M., FRISCH, J., SCHULZ, G., MERTELSMANN, R. & HOELZER, D. (1990*c*). Effects of recombinant human interleukin-3 in patients with myelodysplastic syndromes. *Blood*, **76**, 455–462.

GEISSLER, K., VALENT, P., MAYER, P., LIEHL, E., HINTERBERGER, W., LECHNER, K. & BETTELHEIM. P. (1990). Recombinant human interleukin-3 expands the pool of circulating hematopoietic progenitor cells in primates – synergism with recombinant human granulocyte/macrophage colony-stimulating factor. *Blood*, **75**, 2305–2310.

GIANNI, A.M., SIENA, S., BREGNI, M., TARELLA, C., STERN, A.C., PILERI, A. & BONADONNA, G. (1989). Granulocyte-macrophage colony-stimulating factor to harvest circulating haemopoietic stem cells for autotransplantation. *Lancet*, **2**, 580–585.

GORE, M.E., CALVERT, A.H. & SMITH, L.E. (1987). High dose carboplatin in the treatment of lung cancer and mesothelioma: a phase I dose escalation study. *Eur. J. Cancer Clin. Oncol.*, **23**, 1391–1397.

GRAHAM, G.J., WRIGHT, E.G., HEWICK, R., WOLPE, S.D., WILKIE, N.M., DONALDSON, D., LORIMORE, S. & PRAGNELL, I.B. (1990). Identification and characterization of an inhibitor of haemopoietic stem cell proliferation. *Nature*, **344**, 442–444.

HARLAND, S.J., NEWELL, D.R., SIDDIK, Z.H., CHADWICK, R., CALVERT, A.H. & HARRAP, K.R. (1984). Pharmacokinetics of cis-diammine-1,1-cyclobutane dicarboxylate platinum (II) in patients with normal and impaired renal function. *Cancer Res.*, **44**, 1693–1697.

HAYWARD, J.L., CARBONE, P.P., HEUSON, J.-C., KUMAOKA, S., SEGALOFF, A. & RUBENS, R.D. (1977). Assessment of response to therapy in advanced breast cancer. *Cancer*, **39**,1289–1294.

JUTTNER, C.A., TO, L.B., HAYLOCK, D.N., DYSON, P.G., THORP, D., DART, G.W., HO, J.Q., HORVATH, N. & BARDY, P. (1989). Autologous blood stem cell transplantation. *Transplant. Proc.*, **21**, 2929–2931.

KAYE, S.B., LEWIS, C.R., PAUL, J., DUNCAN, I.D., GORDON, H.K., KITCHENER, H.C. CRUICKSHANK, D.J., ATKINSON, R.J., SOUKOP, M., RANKIN, E.M., CASSIDY, J., DAVIS, J.A., REED, N.S., CRAWFORD, S.M., MACLEAN, A., SWAPP, G.A., SARKAR, T.K., KENNEDY, J.H. & SYMONDS. R.P. (1992). Randomised study of two doses of cisplatin with cyclophosphamide in epithelial ovarian cancer. *Lancet*, **340**, 329–333.

KELLER, J.R., McNIECE, I.K., SILL, K.T., ELLINGSWORTH, L.R., QUESENBERRY, P.J., SING, G.K. & RUSCETTI, F.W. (1990). Transforming growth factor beta directly regulates primitive murine hematopoietic cells proliferation. *Blood*, **75**, 596–602.

KURZROCK, R., TALPAZ, M., ESTROV, Z., ROSENBLUM, M.G. & GUTTERMAN, J.U. (1991). Phase I study of recombinant human interleukin-3 in patients with bone marrow failure. *J. Clin. Oncol.*, **9**, 1241–1250.

LEARY, A.G., YANG, Y.C., CLARK, S.C., GASSON, J.C., GOLDE, D.W. & OGAWA, M. (1987). Recombinant gibbon interleukin 3 supports formation of human multilineage colonies and blast cell colonies in culture: comparison with recombinant human granulocyte-macrophage colony-stimulating factor. *Blood*, **70**, 1343–1348.

LEE, E.J., EGORIN, M.J., VAN ECHO, D.A., COHEN, A.E., TAIT, N. & SCHIFFER, C.A. (1988). Phase I and pharmacokinetic trial of carboplatin in refractory adult leukemia. *J. Natl Cancer Inst.*, **80**, 131–135.

LEVIN, L. & HRYNIUK, W.M. (1987). Dose intensity analysis of chemotherapy regimens in ovarnian carcinoma. *J. Clin. Oncol.*, **5**, 756–767.

LU, L., BRIDDELL, R.A., GRAHAM, C.D., BRANDT, J.E., BRUNO, E. & HOFFMAN, R. (1988). Effect of recombinant and purified human haematopoietic growth factors on *in vitro* colony formation by enriched populations of human megakaryocyte progenitor cells [published erratum appears in Br. J. Haematol. 1988 Dec; 70(4): 503]. *Br. J. Haematol.*, **70**, 149–156.

MANGIONI, C., BOLIS, G., PECORELLI, S., BRAGMAN, K., EPIS, A., FAVALLI, G. GAMBINO, A., LANDONI, F., PRESTI, M. TORRI, W., VASSENA, L., ZANABONI, F. & MARSONI, S. (1989). Randomized trial in advanced ovarian cancer comparing cisplatin and carboplatin [see comments]. *J. Natl Cancer Inst.*, **81**, 1464–1471.

McDONALD, B.R., KIRMANI, S., VASQUEZ, M. & MEHTA, R.L. (1991). Acute renal failure associated with the use of intraperitoneal carboplatin: a report of two cases and review of the literature. *Am. J. Med.*, **90**. 386–391.

MESSNER, H.A., YAMASAKI, K., JAMAL, N., MINDEN, M.M., YANG, Y.C., WONG, G.G. & CLARK, S.C. (1987). Growth of human hemopoietic colonies in response to recombinant gibbon interleukin 3: comparison with human recombinant granulocyte and granulocyte-macrophage colony-stimulating factor. *Proc. Natl Acad. Sci. USA*, **84**, 6765–6769.

METCALF, D., BEGLEY, C.G., JOHNSON, G.R., NICOLA, N.A., LOPEZ, A.F. & WILLIAMSON, D.J. (1986). Effects of purified bacterially synthesized murine multi-CSF (IL-3) on hematopoiesis in normal adult mice. *Blood*, **68**, 46–57.

MORSTYN, G., CAMPBELL, L., SOUZA, L.M., ALTON, N.K., KEECH, J., GREEN, M., SHERIDAN, W., METCALF, D. & FOX, R. (1988). Effect of granulocyte colony stimulating factor on neutropenia induced by cytotoxic chemotherapy. *Lancet*, **1**, 667–672.

MURRAY, N. (1987). The importance of dose and dose intensity in lung cancer chemotherapy. *Semin. Oncol.*, **14**, 20–28.

OHNO, R., TOMONAGA, M., KOBAYASHI, T., KANAMARU, A., SHIRAKAWA, S., MASAOKA, T. OMINE, M., OH, H., NOMURA, T., SAKAI, Y., HIRANO, M., YOKOMAKU, S., YOSHIDA, Y. MUIRA, A.B., MORISHIMA, A., DOHY, H., NIHO, Y., HAMAJIMA, N. & TAKAKU, F. (1990). Effect of granulocyte colony-stimulating factor after intensive induction therapy in relapsed or refractory acute leukemia. *N. Engl. J. Med.*, **323**, 871–877.

OZOLS, R.F., OSTCHEGA, Y., MYERS, C.E. & YOUNG, R.C. (1985). High-dose cisplatin in hypertonic saline in refractory ovarian cancer. *J. Clin. Oncol.*, **3**, 1246–1250.

OZOLS, R.F., OSTCHEGA, Y., CURT, G. & YOUNG, R.C. (1987). High-dose carboplatin in refractory ovarian cancer patients. *J. Clin. Oncol.*, **5**, 197–201.

REIFFERS, J., BERNARD, P.H., MARIT, G., SARRAT, A., FROIDEVAL, J.L., BROUSTET, A. & VEZON, G. (1986). Collection of blood-derived hematopoietic stem cells and application for autologous transplantation. *Bone Marrow Transplant*, [Suppl] **1**, 371–379.

RICHMAN, C.M., WEINER, R.S. & YANKEE, R.A. (1976). Increase in circulating stem cells following chemotherapy in man. *Blood*, **47**, 1031–1039.

SAELAND, S., CAUX, C., FAVRE, C., DUVERT, V., PEBUSQUE, M.J., MANNONI, P. & DE VRIES, J.E. (1989). Combined and sequential effects of human IL-3 and GM-CSF on the proliferation of CD34+ hematopoietic cells from cord blood. *Blood*, **73**, 1195–1201.

SCHMALBACH, T.K. & BORCH, R.F. (1989). Diethyldithiocarbamate modulation of murine bone marrow toxicity induced by cis-diammine(cyclobutanedicarboxylato)platinum (II). *Cancer Res.*, **49**, 6629–6633.

SIENNA, S., BREGNI, M., BRANDO, B., BELLI, N., RAVAGNANI, F., GANDOLA, L., STERN, A.C., LANSDORP, P.M., BONADONNA, G. & GIANNI, A.M. (1991). Flow cytometry for clinical estimation of circulating hematopoietic progenitors for autologous transplanation in cancer patients. *Blood*, **77**, 400–409.

SOCINSKI, M.A., CANNISTRA, S.A., ELIAS, A., ANTMAN, K.H., SCNIPPER, L. & GRIFFIN, J.D. (1988). Granulocyte-macrophage colony stimualting factor expands the circulating haemopoietic progenitor cell compartment in man. *Lancet*, **1**, 1194–1198.

SONODA, Y., YANG, Y.C., WONG, G.G., CLARK, S.C. & OGAWA, M. (1988). Analysis in serum-free culture of the targets of recombinant human hemapoietic growth factors: interleukin 3 and granulocyte/macrophage-colony-stimulating factor are specific for early developmental stages. *Proc. Natl Acad. Sci. USA*, **85**, 4360–4364.

TEICHER, B.A., HOLDEN, S.A., EDER, J.P., BRANN, J.W., JONES, S.M. & FREI, E. (1989). Influence of schedule on alkylating agent cytotoxicity *in vitro* and *in vivo*. *Cancer Res.*, **49**, 5994–5998.

TEN BOKKEL HUININK, W.W., RODENHUIS, S., SIMONETTI, G., DUBBELMAN, R., FRANKLIN, H., DALESIO, O., VERMORKEN, J.B. & McVIE, J.G. (1992). Studies with carboplatin in ovarian cancer: experience of the Netherlands Cancer Institute and GCCG of the European Organization for Research and Treatment of Cancer. *Carboplatin (JM-8). Current Perspectives and Future Directions*. Bunn, P.A., Canetta, R., Ozols, R.F. & Rozencweig, M. (eds).

TO, L.B., SHEPPERD, K.M., HAYLOCK, D.N., DYSON, P.G., CHARLES, P., THORP, D.L., DALE, B.M., DART, G.W., ROBERTS, M.M., SAGE, R.E. & JUTTNER, C.A. (1990). Single high doses of cyclophosphamide enable the collection of high numbers of hemopoietic stem cells from the peripheral blood [see comments]. *Exp. Hematol.*, **18**, 442–447.

WAGEMAKER, G., VAN GILS, F.C., BURGER, H., DORSSERS, L.C., VAN LEEN, R.W., PERSOON, N.L., WIELENGA, J.J., HEENEY, J.L. & KNOL, E. (1990). Highly increased production of bone marrow-derived blood cells by administration of homologous interleukin-3 to rhesus monkeys. *Blood*, **76**, 2235–2241.

WITTES, R.E. (1989). *Manual of Oncologic Therapeutics*. p.627. Lippencott, Philadelphia.